# Genotyping of Single Nucleotide Polymorphisms in DNA Isolated from Serum Using Sequenom MassARRAY Technology

**DOI:** 10.1371/journal.pone.0135943

**Published:** 2015-08-14

**Authors:** Tess V. Clendenen, Justin Rendleman, Wenzhen Ge, Karen L. Koenig, Isaac Wirgin, Diane Currie, Roy E. Shore, Tomas Kirchhoff, Anne Zeleniuch-Jacquotte

**Affiliations:** 1 Department of Population Health, New York University School of Medicine, New York, New York, United States of America; 2 NYU Cancer Institute, New York University Langone Medical Center, New York, New York, United States of America; 3 Department of Environmental Medicine, New York University School of Medicine, New York, New York, United States of America; 4 Radiation Effects Research Foundation, Hiroshima Japan; University of Sydney, AUSTRALIA

## Abstract

**Background:**

Large epidemiologic studies have the potential to make valuable contributions to the assessment of gene-environment interactions because they prospectively collected detailed exposure data. Some of these studies, however, have only serum or plasma samples as a low quantity source of DNA.

**Methods:**

We examined whether DNA isolated from serum can be used to reliably and accurately genotype single nucleotide polymorphisms (SNPs) using Sequenom multiplex SNP genotyping technology. We genotyped 81 SNPs using samples from 158 participants in the NYU Women’s Health Study. Each participant had DNA from serum and at least one paired DNA sample isolated from a high quality source of DNA, i.e. clots and/or cell precipitates, for comparison.

**Results:**

We observed that 60 of the 81 SNPs (74%) had high call frequencies (≥95%) using DNA from serum, only slightly lower than the 85% of SNPs with high call frequencies in DNA from clots or cell precipitates. Of the 57 SNPs with high call frequencies for serum, clot, and cell precipitate DNA, 54 (95%) had highly concordant (>98%) genotype calls across all three sample types. High purity was not a critical factor to successful genotyping.

**Conclusions:**

Our results suggest that this multiplex SNP genotyping method can be used reliably on DNA from serum in large-scale epidemiologic studies.

## Introduction

Single nucleotide polymorphisms (SNPs) have been found to be associated with risk of many complex diseases. For instance, more than 70 susceptibility SNPs have been identified for breast cancer [1–3]. Assessment of gene-environment interactions is a natural next step in research on the etiology of these diseases. Prospective cohort studies have well-annotated data on exposures that are collected prior to diagnosis, thereby limiting the risk of bias. DNA can be isolated from serum or plasma samples collected from these cohorts although it may not be of the high quality/quantity of DNA isolated from other types of biological samples, such as buffy coats or whole blood.

DNA isolated from serum has been used by our group and others to achieve high call frequencies with single SNP Taqman assays (without performing whole genome amplification prior to PCR amplification) in epidemiological studies [4–7]. However, Taqman assays, as with other single-SNP genotyping methods, are time consuming and costly, which limits their usefulness for large-scale studies. We conducted a study to assess the utility of the multiplex Sequenom MassARRAY system (Sequenom Inc., CA) for genotyping SNPs in genomic DNA isolated from serum. Oligonucleotide primers are extended, dependent on the SNP-specific template sequence (PCR product), and MALDI-TOF mass spectrometry is used to differentiate SNP alleles based on the different masses of extension products. We designed this study to compare the frequency of successful genotyping calls and the concordance of calls from paired serum- whole blood DNA collected from subjects in a large prospective cohort study.

## Methods

Between 1985 and 1991, the NYU Women's Health Study (NYUWHS) enrolled 14,274 healthy women aged 35–65 years at a breast cancer screening center. Enrollment in the cohort required donation of 30mL of blood. Blood collection was performed without anti-coagulant; tubes were kept covered at room temperature (25°C) for 15 minutes and then at 4°C for 60 minutes. Tubes were then centrifuged and the serum supernatant was collected and divided into several 1 mL aliquots. Serum samples were stored in polypropylene screw capped tubes at -80°C within 2 hours of collection. For blood samples collected in or after 1988, the cellular precipitates were also partitioned off and stored in polypropylene tubes at -80°C. The remaining blood clots from each sample were stored starting in 1989. Clots were stored immediately at -40°C after serum and cell precipitate removal in two sealable plastic-lined aluminum bags. The NYU Medical Center Institutional Review Board approved this study. Participants completed written informed consent.

This study included samples from 158 NYUWHS participants: 48 women with both serum and clots, 48 with both serum and cell precipitates, and 62 women with all three types of samples, *i*.*e*., serum, clots, and cell precipitates.

DNA was isolated from 200 μL of serum, clots (a section of clot was digested in streptokinase for at least 8 hours prior to DNA isolation), and cell precipitates manually using QIAamp Blood Mini kits from (Qiagen, Inc., Valencia, CA) according to manufacturer protocols. A nanodrop spectrometer was used to estimate DNA concentration and purity using the A260/A280 ratio. Three repeat readings were taken for each DNA sample and the mean of the three measurements was used.

We genotyped 81 SNPs with minor allele frequencies ranging from 2% to 40%. SNPs genotyped in this study were selected for a separate study. Among the 81 SNPs, 57 (70%) were tag SNPs in three vitamin D-related gene regions on three chromosomes, and 24 were SNPs in 19 gene regions on 10 other chromosomes. Approximately 10% of the SNPs were in coding regions, 10% in UTRs, 4% in non-coding regions or upstream of gene regions, and 75% were intronic. Genotyping was performed using multiplex Sequenom technology, using 2 μL per reaction of DNA isolated from serum, clots, and cell precipitates and following standard manufacturer protocols (without whole genome amplification). The genotyping design software generated three multiplex reactions: 29 SNPs (plex1), 28 SNPs (plex 2), and 24 SNPs (plex 3). All genotyping was performed with the MassARRAY iPLEX platform (Sequenom Inc, San Diego, California, USA). Laboratory personnel were blinded to the identity and source of the DNA sample.

Call frequencies, expressed as percentages, were calculated for each SNP. Genotype concordance was assessed by examining groups of paired samples (serum/clot, serum/cell precipitate, and clot/cell precipitate) separately. SNPs that could not be called were excluded from concordance calculations. A call frequency across samples ≥95%, a commonly used standard in epidemiologic studies [8,9], and concordance >98% were considered acceptable.

## Results

Table 1 shows the characteristics of the DNA samples used for this reliability study. Cell precipitates had a median DNA concentration approximately 2-fold higher than that of clots and serum (18.4 μg/ml vs. 8.5 μg/ml and 9.7 μg/ml, respectively). Of the three sample types, only cell precipitates had a majority of samples with an A260/A280 ratio of 1.8–2.0 (72% of cell precipitate DNA samples vs. 29% of clot and 16% of serum DNA samples). Though both clots and cell precipitates are whole blood sources of DNA, isolation of DNA from cell precipitates yielded more DNA with higher purity than clots. Thus, we consider cell precipitate DNA as the highest quality DNA source when comparing with genotyping data based on DNA from serum.

**Table 1:** Spectrophotometer estimated DNA yield and purity, by sample type.

	# of samples	DNA yield, μg/mL, Median (10%, 90%)*	A260/A280		
			<1.8	1.8–2.0	>2.0
DNA Source			n (%)	n (%)	n (%)
Serum	158	9.7 (1.7, 14.4)	101 (64%)	26 (16%)	31 (20%)
Whole Blood Clots	110	8.5 (2.9, 15.1)	40 (36%)	32 (29%)	38 (35%)
Cell Precipitates	110	18.4 (8.3, 49.8)	27 (25%)	78 (71%)	5 (5%)

Note: A260/A280 between 1.8–2.0 indicates good DNA purity

*Based on an averaged measurement of triplicate readings (CVs were ≤7% for A260/A280 for each sample type

As shown in Table 2, about 85% of SNPs had call frequencies ≥95% for clot and cell precipitate sources of DNA vs. 74% for DNA from serum. There were 57 SNPs with call frequencies ≥95% for all three DNA sources (clots, cell precipitates and serum). Out of these 57 high call-frequency SNPs, 54 (95%) showed >98% concordance between serum and clot DNA and 55 SNPs (96%) showed >98% concordance between serum and cell precipitate DNA. Concordance was also >98% for comparisons between clots and cell precipitate DNA (65/68 = 96% of SNPs with call frequencies >95% for both clots and cell precipitates). The call frequencies and concordance data for each SNP are shown in the S1 Table.

**Table 2:** Summary of call frequencies and concordance.

Source of DNA	N of samples	N of SNPs with ≥95% calls	N of SNPs in >98% concordance with serum^a^
Serum	158	60/81 = 74%	
Clots	110	69/81 = 85%	54/57 = 95%
Cell precipitates	110	70/81 = 86%	55/57 = 96%

^a^ Denominator (n = 57) is the number of SNPs with call frequencies ≥95% for all three of the DNA source sample types (clots, cell precipitates, and serum

## Discussion

Our study demonstrates that Sequenom MassARRAY technology can be used to genotype SNPs with a multiplex approach using a small volume of serum DNA. For the SNPs we examined, 74% had call frequencies of 95% or higher (QC criteria commonly used in epidemiological studies [8,9]) in DNA from serum. Of the SNPs with high call frequencies, 95% also had >98% concordance with genotypes from clot and cell precipitate DNA, indicating that DNA from low quantity/quality serum sources can be reliably genotyped. The proportion of SNPs with ≥95% successful calls for serum DNA (74%) was only slightly lower than that observed for DNA from clots (85%) and cell precipitates (86%), and concordance was very high for SNPs with high call frequencies (95% of these SNPs had concordance >98%).

Only one other study examined the utility of multiplex Sequenom for genotyping DNA isolated from serum. It concluded that the genotyping using Sequenom was not reliable, contrary to genotyping using Taqman methods [4]. In that study, DNA was isolated using Qiagen kits for 50 paired samples from white blood cells and serum. Forty-eight SNPs were genotyped (number of plex reactions not given) and overall call frequencies were described in general terms as poor with high discordance for serum DNA. Call frequencies and concordance were also low for a second high-throughput (SNPlex) method that was used to genotype the same samples in a different laboratory. Genotypes obtained from serum DNA across laboratories differed for many SNPs. DNA yields (from samples from the same cohort) were 10–474 ng/mL, substantially lower than our estimated yields, but the investigators noted that concentrations may have been underestimates based on PCR results and that these measurements were done in samples after ethyl acetate and hexane extraction (which is a non-standard treatment of samples prior to DNA analysis). The different observations of that prior study compared to ours may be due to several factors, besides overall DNA quantities: 1) Difference in the number of SNPs included in each multiplex reaction: Higher plexing could reduce genotyping quality; 2) Serum separation procedures (i.e., the use of separator tubes vs. non-separator tubes in our study), which may impact the quantity or quality of serum DNA; 3) Advancements in Sequenom SNP assay designs since 2008 which have made Sequenom technology more accommodating to lower concentration and/or quality DNA; and 4) Difference in SNPs selection between studies, and hence different pools of SNPs constituting the multiplex reactions.

A DNA sample with an A260/A280 ratio of 1.8–2.0 is considered to have low contamination (e.g. by proteins and carbohydrates). Only 29% of clots and 16% of serum DNA had values within this range. It is possible that different DNA isolation protocols/kits, and, for serum, a volume larger than 200 μL, would have led to better results. We were not able to compare different approaches for DNA isolation because the serum samples collected in the NYUWHS (and in other long-term cohorts) are very precious, and the minimal necessary volume is allocated for each assay. It should be noted though that we achieved successful genotyping calls and high concordance for over 70% of SNPs, despite the low percentage of samples within the 1.8–2.0 range. Further, excluding DNA samples with A260/A280 ratios below 1.8 or above 2.0 did not result in appreciable differences in the percentage of SNPs with acceptable call frequencies (data not shown). Since exclusion of samples (and therefore of participants) results in loss of power and possibly bias in epidemiologic studies, it does not appear that it would be a good strategy to exclude from the statistical analysis of these studies samples falling outside the 1.8–2.0 range for the A260/A280 ratio. High purity of DNA does not appear to be a critical factor to successful genotyping using Sequenom iPLEX.

Genome wide association studies (GWAS) and other large targeted genotyping scans, apply relatively strict criteria for exclusion of samples with low genotype call frequencies across all SNPs [10,11]. Excluding samples in our data set with call frequencies <90% across all SNPs resulted in the removal of 6 clots (5%), 7 cell precipitates (6%), and 47 serum samples (30% of all serum samples in the study, data not shown). While these exclusions increased the number of SNPs with call frequencies ≥95% for serum DNA (from 74% to 84%), at the same time they reduced the total number of DNA serum samples by ~30%. Concordance was similar for all SNPs whether or not these samples were included. Our findings, therefore, suggest that excluding samples with low call frequencies would not substantially reduce the rate of genotype errors, and would result in the loss of a large number of subjects for studies in which serum is the source of DNA.

With the exception of one, all the SNPs with a high call frequency (≥95%) in serum DNA samples were also highly concordant (≥98%) with DNA from cell precipitates (considered as a high quality reference in our study comparison). This suggests that applying a stringent call-frequency criterion leads to the exclusion of SNPs with unreliable genotype calls. This result is consistent with the high correlation between miscalls and no calls observed by others [12] and suggests that the multiplex Sequenom platform can be successfully used for genotyping DNA extracted from serum. As is done routinely, SNPs that do not meet QC criteria could be replaced with SNPs in high linkage disequilibrium that might be more easily genotyped with Sequenom technology or alternatively, genotyped using single SNP assays, such as Taqman. Such SNPs could be identified by conducting a pilot study prior to genotyping case-control study samples.

Isolation of DNA from 200 μL of serum (average yield ~425ng of DNA suspended in 50 μL buffer, using the Qiagen blood kit and protocol) provides a sufficient amount of DNA to genotype up to 1,000 SNPs using Sequenom (2 μL DNA for each of 25 reactions, 40-plex each = 1000 SNPs). Assuming our results are broadly applicable to other SNP loci, a similarly conducted genotyping experiment from serum DNA would yield approximately 75% of SNPs having >95% call frequencies (and also ≥98% concordance).

## Conclusion

Considering that for many diseases, GWAS and other large targeted genotyping studies have identified susceptibility SNPs which are expected to number at most in the hundreds, and that many SNPs can be tagged by other SNPs in high LD should the assay not work for certain SNPs, our study shows that the iPLEX Sequenom platform appears to be a useful tool for SNP genotyping in epidemiologic studies that have serum samples as the only source of DNA for their participants.

## Supporting Information

S1 Table Call frequency and concordance by SNP, all samples. (DOCX)
