# Supplementary material for: Genotyping of Single Nucleotide Polymorphisms in DNA Isolated from Serum Using Sequenom MassARRAY Technology
Source: PLoS One. 2015 Aug 14;10(8):e0135943. doi: 10.1371/journal.pone.0135943 (PMC4537187; doi:10.1371/journal.pone.0135943)
Supplement: S1 Table — (DOCX) [file pone.0135943.s001.docx]

S1 Table: Call frequency and concordance by SNP, all samples

|  |  | Call frequency (percent) | | | Minor allele frequency in serum | Percent Concordance | | |
| --- | --- | --- | --- | --- | --- | --- | --- | --- |
| SNP | Batch | Clot  N=110 | Cell Precipitate  N=110 | Serum  N=158 |  | Serum/Cell Precipitate pairs concordance | Serum/Clot pairs concordance | Clot/Cell Precipitate pairs concordance |
| Call frequency ≥ 95% for all sample types (clot, cell precipitate, and serum) | | | | | | | | |
| 1. rs12004786 | A | 98% | 98% | 97% | 0.159 | 100% | 100% | 100% |
| 1. rs13281615 | A | 98% | 98% | 96% | 0.375 | 100% | 99% | 100% |
| 1. rs1801133 | A | 98% | 95% | 96% | 0.357 | 100% | 100% | 100% |
| 1. rs2239180 | A | 99% | 96% | 96% | 0.116 | 100% | 100% | 100% |
| 1. rs2245153 | A | 98% | 97% | 96% | 0.241 | 100% | 100% | 100% |
| 1. rs2296241 | A | 98% | 97% | 95% | 0.349 | 98% | 100% | 98% |
| 1. rs2408876 | A | 99% | 98% | 97% | 0.326 | 100% | 100% | 100% |
| 1. rs2426498 | A | 99% | 97% | 97% | 0.122 | 100% | 100% | 100% |
| 1. rs2762926 | A | 98% | 98% | 97% | 0.284 | 100% | 100% | 100% |
| 1. rs2762934 | A | 98% | 98% | 97% | 0.218 | 100% | 99% | 100% |
| 1. rs2853564 | A | 99% | 98% | 97% | 0.311 | 100% | 100% | 100% |
| 1. rs2981579 | A | 98% | 95% | 97% | 0.362 | 100% | 100% | 100% |
| 1. rs3118526 | A | 98% | 98% | 97% | 0.119 | 100% | 100% | 100% |
| 1. rs34312136 | A | 98% | 97% | 97% | 0.353 | 100% | 100% | 100% |
| 1. rs34857233 | A | 98% | 95% | 97% | 0.067 | 100% | 100% | 100% |
| 1. rs3787557 | A | 99% | 98% | 97% | 0.135 | 100% | 100% | 100% |
| 1. rs3819545 | A | 98% | 95% | 96% | 0.304 | 100% | 100% | 100% |
| 1. rs4328262 | A | 98% | 98% | 97% | 0.385 | 100% | 100% | 100% |
| 1. rs549438 | A | 99% | 98% | 97% | 0.365 | 100% | 100% | 100% |
| 1. rs9729 | A | 99% | 98% | 97% | 0.323 | 100% | 100% | 100% |
| 1. rs1045485 | B | 99% | 98% | 98% | 0.067 | 100% | 100% | 100% |
| 1. rs1045570 | B | 99% | 98% | 97% | 0.160 | 100% | 98% | 100% |
| 1. rs10741657 | B | 99% | 98% | 97% | 0.267 | 100% | 100% | 100% |
| 1. rs10875695 | B | 98% | 97% | 97% | 0.273 | 100% | 100% | 100% |
| 1. rs11168267 | B | 99% | 97% | 98% | 0.096 | 100% | 100% | 100% |
| 1. rs11185644 | B | 99% | 98% | 97% | 0.261 | 100% | 100% | 100% |
| 1. rs11185659 | B | 99% | 98% | 97% | 0.224 | 100% | 100% | 100% |
| 1. rs11574044 | B | 99% | 98% | 98% | 0.199 | 100% | 99% | 100% |
| 1. rs12006409 | B | 97% | 95% | 96% | 0.102 | 100% | 100% | 100% |
| 1. rs1562430 | B | 99% | 98% | 97% | 0.372 | 100% | 100% | 100% |
| 1. rs1801131 | B | 99% | 98% | 97% | 0.272 | 100% | 100% | 100% |
| 1. rs2107301 | B | 98% | 95% | 97% | 0.262 | 100% | 100% | 100% |
| 1. rs2189480 | B | 97% | 96% | 96% | 0.282 | 100% | 100% | 100% |
| 1. rs2248359 | B | 99% | 97% | 97% | 0.358 | 99% | 100% | 100% |
| 1. rs529858 | B | 99% | 98% | 97% | 0.028 | 100% | 100% | 100% |
| 1. rs757343 | B | 99% | 97% | 98% | 0.158 | 100% | 99% | 100% |
| 1. rs927650 | B | 99% | 98% | 97% | 0.334 | 99% | 99% | 100% |
| 1. rs10776909 | C | 100% | 98% | 97% | 0.214 | 100% | 100% | 100% |
| 1. rs12785878 | C | 100% | 97% | 99% | 0.382 | 100% | 100% | 100% |
| 1. rs1570669 | C | 100% | 98% | 98% | 0.349 | 100% | 99% | 100% |
| 1. rs17251221 | C | 97% | 97% | 96% | 0.207 | 95% | 96% | 96% |
| 1. rs1799945 | C | 100% | 97% | 98% | 0.099 | 100% | 100% | 100% |
| 1. rs1800562 | C | 100% | 98% | 99% | 0.017 | 100% | 100% | 100% |
| 1. rs1805343 | C | 100% | 98% | 99% | 0.393 | 100% | 100% | 100% |
| 1. rs1907362 | C | 96% | 98% | 97% | 0.063 | 100% | 100% | 100% |
| 1. rs2228570 | C | 100% | 97% | 97% | 0.338 | 100% | 99% | 100% |
| 1. rs2239182 | C | 100% | 97% | 99% | 0.342 | 100% | 100% | 100% |
| 1. rs2244719 | C | 100% | 98% | 98% | 0.307 | 100% | 99% | 100% |
| 1. rs2762927 | C | 100% | 98% | 99% | 0.299 | 100% | 100% | 100% |
| 1. rs2762941 | C | 100% | 98% | 99% | 0.298 | 100% | 100% | 100% |
| 1. rs3811647 | C | 100% | 98% | 97% | 0.288 | 100% | 100% | 100% |
| 1. rs3890733 | C | 100% | 98% | 97% | 0.309 | 99% | 98% | 98% |
| 1. rs4237856 | C | 100% | 98% | 98% | 0.210 | 100% | 99% | 100% |
| 1. rs4415084 | C | 100% | 98% | 99% | 0.344 | 100% | 100% | 100% |
| 1. rs4842196 | C | 100% | 97% | 99% | 0.334 | 100% | 100% | 100% |
| 1. rs6013897 | C | 100% | 98% | 98% | 0.255 | 100% | 100% | 100% |
| 1. rs889312 | C | 100% | 98% | 99% | 0.286 | 100% | 99% | 100% |
| Call frequency ≥ 95% for clots and cell precipitates but not serum | | | | | | | | |
| 1. rs11568820 | A | 98% | 98% | 94% | 0.319 | 100% | 100% | 100% |
| 1. rs2239186 | A | 98% | 95% | 91% | 0.174 | 100% | 100% | 100% |
| 1. rs2762929 | A | 95% | 95% | 84% | 0.281 | 99% | 100% | 100% |
| 1. rs3803662 | A | 98% | 97% | 94% | 0.342 | 100% | 100% | 100% |
| 1. rs1544410 | B | 97% | 95% | 72% | 0.320 | 100% | 100% | 100% |
| 1. rs2180341 | B | 96% | 96% | 86% | 0.227 | 100% | 99% | 100% |
| 1. rs2208588 | B | 98% | 98% | 91% | 0.379 | 100% | 100% | 100% |
| 1. rs3787555 | B | 98% | 98% | 94% | 0.247 | 98% | 99% | 100% |
| 1. rs4240705 | B | 95% | 96% | 79% | 0.337 | 100% | 100% | 100% |
| 1. rs490361 | C | 99% | 98% | 92% | 0.301 | 100% | 100% | 100% |
| 1. rs6097801 | C | 95% | 98% | 72% | 0.183 | 93% | 94% | 100% |
| Call frequency ≥ 95% for serum but not clots or cell precipitates | | | | | | | | |
| 1. rs1110102 | B | 90% | 93% | 96% | 0.133 | 100% | 99% | 100% |
| 1. rs1790349 | B | 89% | 82% | 95% | 0.115 | 99% | 100% | 100% |
| 1. rs4917348 | C | 88% | 81% | 95% | 0.251 | 100% | 99% | 100% |
| Call frequency <95% for serum and either clots or cell precipitates | | | | | | | | |
| 1. rs13387042 | A | 72% | 96% | 0% | ND | ND | ND | 100% |
| 1. rs2209314 | B | 77% | 95% | 11% | 0.244 | 100% | 100% | 100% |
| 1. rs2181874 | C | 97% | 94% | 89% | 0.301 | 96% | 99% | 100% |
| Call frequency <95% for all three sample types (serum, clots, cell precipitates) | | | | | | | | |
| 1. rs1155563 | A | 93% | 94% | 87% | 0.219 | 93% | 93% | 93% |
| 1. rs1540339 | A | 45% | 81% | 0% | ND | ND | ND | 96% |
| 1. rs3118536 | A | 87% | 89% | 75% | 0.188 | 99% | 100% | 100% |
| 1. rs3817198 | A | 22% | 8% | 0% | ND | ND | ND | ND |
| 1. rs2239179 | B | 76% | 74% | 79% | 0.060 | 99% | 99% | 100% |
| 1. rs3118523 | B | 45% | 44% | 58% | 0.159 | 50% | 72% | 92% |
| 1. rs3847987 | B | 11% | 11% | 15% | 0.287 | 33% | 100% | ND |
